# Still careless: Findings from a cross sectional study of young pedestrians’ risky road crossing behaviors

**Published:** 2019-08

**Authors:** Mina Hashemiparast, Ali Montazeri

**Affiliations:** ^*a*^Department of Public Health, Maragheh University of Medical Sciences, Maragheh, Iran.; ^*b*^Population Health Research Group, Health Metrics Research Center, Institute for Health Sciences Research, ACECR, Tehran, Iran.

## Abstract

**Background:**

Pedestrian vehicle-collision is known as one of a higher risk of traffic injuries evidently most occurs at younger age. This study aimed to investigate road crossing behaviors in potential risky situations, attitude towards traffic rules, and perceived risk and severity of traffic accident among young pedestrians in Iran.

**Methods:**

This was a population-based study among 562 young people aged 18 to 25 years living in Tehran, Iran. Survey data were collected using a self-administered questionnaire comprising items on self-reported risky road crossing behaviors, attitude towards traffic rules and regulations, perceived risk, perceived severity of involvement in an accident.

**Results:**

All 562 young individuals were studied. Participants who were involved in a vehicle-collision (n = 100, 17.8%) reported less safety behaviors compared with those who did not experience accident. They also had a negative attitude toward traffic rules and regulations (p=0.001); low perceived risk of road traffic accidents (p=0.02), hazards and the potential severity of their consequences (p=0.004) compared with those who did not experience accident.

**Conclusions:**

The findings indicated that young pedestrians with previous experience of vehicle-collision were still careless and repeated their risky behaviors in crossing the street. Hence, previous unpleasant experience of vehicle-collision could not prevent young people from risky road crossing behaviors. The findings suggest that as well as traditional educational interventions use of health behavior theories to change attitudes towards risk, feelings of invulnerability and perception of vehicle collision severity has to be exploited. It also may be useful to try to focus on the people who have been involved in an accidents rather than relying on public population.

**Keywords:**

Pedestrian, Road-crossing, Traffic injuries, Vehicle-collision history

